# Environmental epigenetics: prospects for studying epigenetic mediation of exposure–response relationships

**DOI:** 10.1007/s00439-012-1189-8

**Published:** 2012-06-28

**Authors:** Victoria K. Cortessis, Duncan C. Thomas, A. Joan Levine, Carrie V. Breton, Thomas M. Mack, Kimberly D. Siegmund, Robert W. Haile, Peter W. Laird

**Affiliations:** 1 Department of Preventive Medicine, Keck School of Medicine, University of Southern California, USC Norris Comprehensive Cancer Center, 1441 Eastlake Avenue, Los Angeles, CA 90089 USA; 2 Department of Preventive Medicine, Keck School of Medicine, University of Southern California, 2001 N. Soto St., SSB-202F, Los Angeles, CA 90089-9234 USA; 3 Department of Preventive Medicine, Keck School of Medicine, University of Southern California, 2001 N. Soto St., Los Angeles, CA 90089-9234 USA; 4 Departments of Surgery, Biochemistry and Molecular Biology, Keck School of Medicine, University of Southern California, USC Norris Comprehensive Cancer Center, Epigenome Center, 1441 Eastlake Avenue, Los Angeles, CA 90089-9601 USA

## Abstract

Changes in epigenetic marks such as DNA methylation and histone acetylation are associated with a broad range of disease traits, including cancer, asthma, metabolic disorders, and various reproductive conditions. It seems plausible that changes in epigenetic state may be induced by environmental exposures such as malnutrition, tobacco smoke, air pollutants, metals, organic chemicals, other sources of oxidative stress, and the microbiome, particularly if the exposure occurs during key periods of development. Thus, epigenetic changes could represent an important pathway by which environmental factors influence disease risks, both within individuals and across generations. We discuss some of the challenges in studying epigenetic mediation of pathogenesis and describe some unique opportunities for exploring these phenomena.

## Background

The field of epigenetics grew from attempts, beginning over 70 years ago, to understand mechanisms whereby multiple cellular phenotypes arise from a single genotype during the complex process of developmental morphogenesis termed epigenesis. The term “epigenetics” was initially reserved for mechanisms by which phenotypic state, as determined by differential gene expression, could be stably retained through cell division by non-genetic factors. Various mechanisms have been proposed to have the potential to encode this phenotypic information; these include enzymatic methylation of cytosine bases (DNA methylation), post-translational modification of tail domains of histone proteins (histone modifications) and associated nucleosome positioning or chromatin remodeling, non-coding RNAs, and transcription factor regulatory networks (Ptashne 2007). Epigenetic marks established by each of these processes are often shared within a cell lineage; however, whether all persisting epigenetic marks satisfy requirements for stable transmission through cell division or some are merely reestablished from other information following mitosis remains a vigorously debated question.

The term epigenetics has more recently been used in the scientific literature to describe various unspecified non-genetic mechanisms influencing phenotype. This broader usage emerged from mouse studies addressing transgenerational nutritional effects on phenotype, as well as human studies of phenotypic differences between monozygotic twins. In the popular press “epigenetics” has become almost synonymous with nutritional and environmental influences on gene expression. Thus, while “epigenetics” initially referred to largely self-contained developmental processes, it has come to describe environmental influences on phenotypic readout of genotypes. This semantic evolution has caused confusion and controversy regarding the meaning of “epigenetics” at a time of intensified interest in the possible role of epigenetic mechanisms in disease. In this review we define as *epigenetic processes* those that stably affect gene expression through mechanisms not involving the primary nucleotide sequence, and *epigenetic state* as the configuration of chromatin and DNA marks utilized by these processes. By contrast, *genetic state* is widely understood to refer to the primary nucleotide sequence itself, while *genetic processes* maintain or change nucleotide sequence.

Epidemiologic research addressing epigenetic mechanisms as mediators of environmental exposures on disease risk is constrained by important ethical considerations. These often preclude both experimental exposure to candidate environmental causes, and invasive collection of cell types of greatest developmental and functional relevance to disease processes. Inquiry has therefore progressed largely by integrating information about biological mechanisms obtained in model systems with observational data provided by humans. To address the current state and future promise of this research, we undertook this review with two goals: to illustrate the potential of epigenetic processes to mediate exposure–phenotype relationships and to discuss study design and statistical analysis methods needed to investigate such mechanisms in relation to origins of human disease. We begin by discussing genetic, developmental, and environmental determinants of epigenetic state in human and model systems, then describe some of the diverse data implicating epigenetic mechanisms in various human diseases, both within individuals and across generations. We conclude by discussing technical challenges, suggesting promising opportunities for epidemiologic research in environmental epigenetics, and offering some thoughts about translational significance and future directions of this field.

## Determinants of epigenetic state

Epigenetic mechanisms work in concert to influence the potential for gene expression at myriad locations throughout the genome. The resulting epigenetic state of the genome, termed *epigenome*, varies by cell type. Considering the tremendous diversity of epigenetic marks, which include dozens of different post-translational histone modifications and more than 50 million sites of potential DNA methylation in a diploid human genome (and thus >2^50M^ possible epigenotypes!), it seems that no two human cells would have identical epigenomes. Indeed, within each individual there are many epigenomes, and these change over time as a consequence of both normal developmental and pathological processes, as well as environmental exposures and random drift. Despite this potential for considerable variability of epigenetic patterns within and between individuals, there can also be remarkable consistency. In a study of 11 tissues in 6 autopsies, DNA methylation patterns in a highly selected set of loci were found to be highly conserved, with intraclass correlations of 0.85 across tissues within individuals and 0.83 across individuals within tissues (Byun et al. 2009). The authors interpreted these patterns as revealing different sets of person-specific and tissue-specific differentially methylated genes, anticipating subsequently observed differential genetic and acquired determination (Waterland et al. 2010).

DNA methylation has been the epigenetic mark most extensively measured in epidemiologic research for numerous reasons. It is of fundamental biological interest owing to its unambiguously stable transmission during cell division. It also has practical advantages: as a chemically stable covalent change to the DNA itself, DNA methylation is the only epigenetic mark that survives the DNA extraction and purification that is routine in molecular sample processing, and it can endure decades of archival sample storage (Kristensen et al. 2009).

### Genetic influences

Nucleotide sequence is a primary determinant of epigenetic state, clearly evident from the distribution of epigenetic marks across the genome, determined in part by direct effects of G:C content and CpG (cytosine-phosphate-guanine dinucleotide) density (Tanay et al. 2007; Thomson et al. 2010). Additional genetic influences include proximity to repetitive elements such as Alu and LINE1 (Estecio et al. 2010), nuclear architecture (Berman et al. 2011), and binding sequences for transacting proteins (Bell et al. 2011b; Weth and Renkawitz 2011). Motif searches and screening strategies have identified sequence elements that predispose to particular epigenetic states (Feltus et al. 2006; Ideraabdullah et al. 2011; Keshet et al. 2006; Lienert et al. 2011).

Several lines of evidence indicate that genetic polymorphisms can affect epigenetic state. Greater differences were observed between dizygotic co-twins than between monozygotic co-twins in two forms of epigenetic state: skewed patterns of X-inactivation, and DNA methylation at differentially methylated regions of the imprinted *IGF2/AH19* locus (Wong et al. 2011; Heijmans et al. 2007; Ollikainen et al. 2010). Extensive DNA methylation analyses in a multigenerational family revealed that epiallelic similarity was greater among first-degree relatives than among more distantly related family members. In the same study, analyses addressing both genetic variation and DNA methylation identified widespread occurrence of allele-specific DNA methylation (ASM) that was associated with polymorphic nucleotides located near the DNA methylation site, but not parent of origin. Authors of this report concluded that the majority of such ASM events depend on *cis*-acting DNA sequence (Gertz et al. 2011). Such ASM events have yet to be characterized in large population-based studies, but more modest studies addressing heterozygous non-imprinted loci have identified widespread ASMs associated with nearby genotypic polymorphisms in DNA from multiple tissue types (Kerkel et al. 2008; Tycko 2010; Schalkwyk et al. 2010), as well as allele-specific chromatin structure and transcription factor binding in lymphoblastoid cell DNA (McDaniell et al. 2010, reviewed in Birney et al. 2010). Presumed transgenerational inheritance of epigenetic changes (“epimutations”) in the *MLH1* (Suter et al. 2004) and *MSH2* (Chan et al. 2006) mismatch repair genes, both associated with colorectal cancer, were also traced to germline genetic variation. In the case of the *MSH2* epimutation, deletion of a gene immediately upstream of the *MSH2* gene causes transcription to run through the *MSH2* promoter, causing somatic hypermethylation and gene silencing (Ligtenberg et al. 2009). The *MLH1* epimutation was found to be caused by a polymorphism in the 5′ UTR of the *MLH1* gene, reducing transcriptional activity, and predisposing to aberrant somatic DNA methylation in each generation (Hitchins et al. 2011).

### Developmental programming of the epigenome

In successful mammalian reproduction, the single-cell zygote gives rise to an organism with hundreds of cell types. These diverse cellular phenotypes arise from the same shared genomic sequence by control of the subset of genes expressed in each cell type. Cellular differentiation is tightly linked to extensive erasure and establishment of lineage-specific epigenetic marks, a process termed epigenetic reprogramming. Relatively detailed descriptions of DNA methylation in developing tissues have been carried out in the mouse, which serves as a model of epigenetic reprogramming in mammalian development (Trasler 2009).

**Fig. 1:**
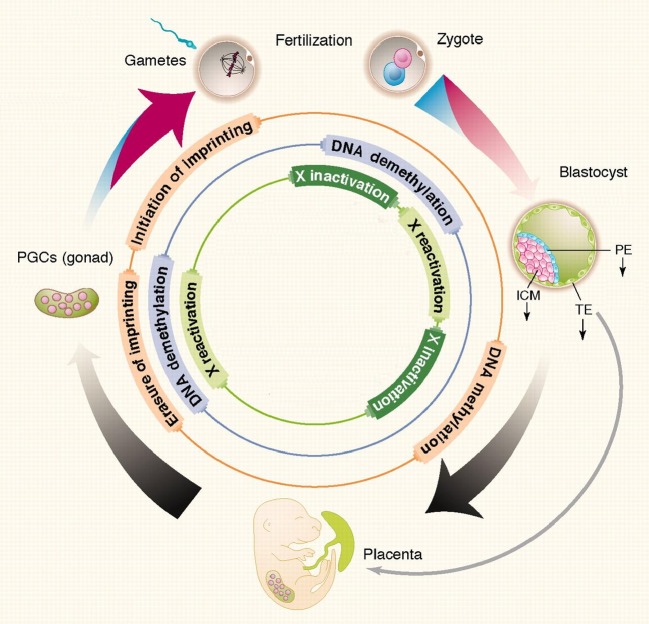
Reprogramming of DNA methylation in the zygote, early embryo, and primordial germ cells. *Thickness of the* *outer arrows* indicates levels of DNA methylation. *Red* maternal genome, *blue* paternal genome, *black* diploid genome. Embryonic lineages arise from cells of the inner cell mass (ICM), the placenta and extraembryonic membranes from trophectoderm cells, and the germ cell lineage from primordial germ cells following their determination from proximal epiblast. *Inner circles* indicate developmental stages when key elements of epigenetic programming are thought to occur (Adapted from Feng et al. 2010)

At fertilization, reprogramming begins with extensive erasure of methyl marks in DNA of the paternal (sperm-derived) DNA, followed by more general loss of methyl marks in the zygote and embryo during cleavage divisions, while sparing parent-of-origin specific imprints. By the blastocyst stage, de novo DNA methylation distinguishes inner cell mass cells (from which embryonic lineages arise to create fetal structures) from relatively hypomethylated trophectoderm cells (from which extra-embryonic lineages arise to create transient structures, including placenta) (Fig. 1).

Germ cell lineage specification begins in cells of the proximal epiblast, and involves a second extensive erasure of DNA methylation that removes parental imprint marks (Fig. 1). Thereafter, the germ line develops in a sexually dimorphic fashion. New DNA methyl marks are established over many stages, extending through sexual maturity in accordance with the sex of the developing individual. At this time, the sex-specific imprint marks that govern parent-of-origin specific expression of imprinted genes in the subsequent generation are established (Faulk and Dolinoy 2011).

Developmental reprogramming can result in dramatic epigenetic differences between the two alleles. The association between mono-allelic gene expression and DNA methylation has long been recognized, both in the context of X-inactivation in females (Boggs et al. 2002; Sharp et al. 2011) and in parent-of-origin determined genomic imprinting (Ferguson-Smith 2011), but now also in the mono-allelic expression of non-imprinted autosomal loci (Harris et al. 2010; Tarutani and Takayama 2011).

Further resetting of epigenetic marks accompanies differentiation of many specialized cell types of the body as well as placenta and other transient structures during pregnancy, and subsequent development of body structures during various postnatal stages of development. Chromatin states that arise during development can affect the propensity to subsequent epigenetic change. An example of this is the predisposition of polycomb-repressive complex occupied genes in stem cells to the acquisition of DNA methylation abnormalities in aging and cancer (Ohm et al. 2007; Schlesinger et al. 2007; Teschendorff et al. 2010; Widschwendter et al. 2007).

### Environmental influences

Multiple differences in gene expression, presumably reflecting intrauterine epigenetic differences, have been identified in several tissues from newborn identical twins (Gordon et al. 2011). The global methylation pattern of individuals changes with increasing age (Bjornsson et al. 2008), as does the difference in global methylation between MZ twin pairs (Fraga et al. 2005). Genetically identical MZ twins show some epigenetic discordance at birth, as indicated by gene expression discordance (Gordon et al. 2011). Even over the first decade of life (Wong et al. 2010), and as aging adults (Talens et al. 2010), MZ twins acquire additional differences in epigenetic state, which may partly reflect different exposure histories, as would be expected if environmental exposures influence epigenetic state. However, stochastic drift in epigenetic state and related consequences such as mono-allelic expression described in the previous section is likely responsible for much of the observed divergence. Therefore, other forms of data (discussed below) are needed to determine what type of exposures may influence epigenetic state and the extent of resulting changes.

### Experimental studies

The most direct evidence suggesting that ambient exposures may influence epigenetic state is experimental. In vitro studies have demonstrated associations of DNA methylation with various metals (Dolinoy et al. 2007b; Wright and Baccarelli 2007). In the in vivo setting, prenatal protein restriction is associated with hypomethylation of the glucocorticoid receptor (*GR*) and *PPARα* gene promoter regions in rat liver (Lillycrop et al. 2005), changes that were prevented by folic acid supplementation (Lillycrop et al. 2005) and which were transmitted to the F2 generation (Burdge et al. 2007). Plagemann et al. (2009) found hypermethylation in the promoter of the anorexigenic gene for proopiomelanocortin in rats overfed as neonates. Whether this change could be transmitted to offspring was not assessed. DNA from sperm of mice exposed to steel plant air was found to be persistently hypermethylated long after exposure ended (Yauk et al. 2008). Additionally, maternal nurturing behavior has been shown to modify methylation at individual CpG sites in the ngf1a binding region of the *GR* gene in the hippocampus of the offspring (Weaver et al. 2004), an epigenetic modification that persisted both into adulthood to modify response to stress, and into the F2 generation.

### Human studies

Christensen and Marsit (2011) and Terry et al. (2011) have provided comprehensive reviews of environmental influences on epigenetic state in humans. Here we note exposure periods of particular interest and several examples of environmental exposures reportedly associated with epigenetic state of specific human cell types.

The epigenome may be most vulnerable to environmental insults during periods of extensive epigenetic reprogramming, which may in theory be disrupted by exposures that interfere with any process that governs reprogramming. Periods of particular vulnerability may therefore include the early stages of embryonic development mentioned above. Childhood is also proposed as a period of vulnerability, especially in the germline of females, since oocytes remain in a haploid de-methylated state until puberty, so environmental insults may potentially disturb the epigenetic state of the oocyte for many years (Faulk and Dolinoy 2011), with potential implications for both fertility and initial epigenetic state of offspring of an exposed female. Somatic changes to DNA methylation may also result from environmental exposures in adults, as have been observed in aging and disease processes such as cancer described in the next section.

#### Energy and nutrient intake

Significant epigenetic changes in the *IGF2* gene have been documented in those prenatally exposed to severe caloric restriction during the Dutch hunger winter of World War II (Heijmans et al. 2008). Hughes et al. (2009) additionally found that those most likely to be exposed to this famine during adolescence or young adulthood had a significantly decreased risk of developing colorectal cancers (CRC) characterized by the CpG island methylator phenotype (CIMP), suggesting a role for early life exposures in CIMP-specific CRC pathogenesis.

Folates are the major source of the methyl groups used for DNA and histone methylation. One study of folates and other one-carbon nutrients reported a differential effect of folate on the risk of the CIMP CRC subset compared to the non-CIMP subset (Van Guelpen et al. 2010), while two other studies did not (Slattery et al. 2006; van den Donk et al. 2007). Most studies of the microsatellite instability high subset, characterized by hypermethylation of the *MLH1* gene promoter region and CIMP (Weisenberger et al. 2006), have yielded similarly negative results (Eaton et al. 2005; Schernhammer et al. 2008; Slattery et al. 2001; Wark et al. 2005). On the other hand, in some studies the association between intake of alcohol (which degrades folates) and CRC risk has been reported to be greater in MSI-H and CIMP tumors (Diergaarde et al. 2003; Eaton et al. 2005; Slattery et al. 2001).

Micro-RNAs (miRNAs) are very short non-coding RNA molecules that can downregulate protein-coding genes by destabilizing mRNAs or blocking translation. The possibility that exogenous microRNA consumed in food may epigenetically regulate gene expression has emerged from recent studies demonstrating the presence of plant-derived miRNAs in sera of humans and other mammals (Zhang et al. 2012). One of these plant microRNAs, MIR168a, which was demonstrated to be only of plant origin in control mice, binds coding sequence of the mammalian *LDLRAP1* gene in vitro. Functional consequences in mammalian systems were demonstrated experimentally, as MIR168a administered in vitro and during in vivo feeding studies decreased expression of the protein product of *LDLRAP1*. This line of research suggests novel epigenetic mechanisms whereby diet may modify risk of human disease.

#### Air pollution

Emerging evidence suggests that air pollutants can influence epigenetic changes, including DNA methylation as well as up- or down-regulation of miRNAs (Jardim 2011). In human epidemiologic studies, PM_2.5_ and PM_10_ exposures are associated with hypomethylation of Alu and/or LINE1 elements in leukocytes and buccal cells (Baccarelli et al. 2009; Bollati and Baccarelli 2010; Madrigano et al. 2011; Salam et al. 2012; Tarantini et al. 2009), as well as altered DNA methylation in *NOS2A,* a gene involved in production of nitric oxide (Salam et al. 2012; Tarantini et al. 2009). Living in highly polluted cities (high PM and ozone) is also associated with hypermethylation of *FOXP3* in regulatory T cells (Nadeau et al. 2010), while neonates who were prenatally exposed to polyaromatic hydrocarbon (PAH) had hypermethylated *ACSL3* in DNA of umbilical cord white blood cells (Perera et al. 2009); notably, both of these genes are involved in asthma pathogenesis. PAHs are also associated with hypermethylation of LINE1 and Alu (Pavanello et al. 2009; Perera et al. 2009). More limited evidence is emerging to suggest that air pollution is associated with changes in miRNA expression (Bollati et al. 2010; Jardim 2011), and adverse effects of air pollution constituents may be modified by variant alleles of genes involved in miRNA processing (Wilker et al. 2010).

#### Tobacco smoke

Fetal exposure to maternal smoking during pregnancy (PTS) is associated with reduced methylation of several repeated sequences, including Sat2 (Flom et al. 2011), Alu, and LINE1 among children with the *GSTM1* null genotype (Breton et al. 2009). PTS exposure is also associated with increased DNA methylation in specific genes, such as *AXL* and *PTPRO* (Breton et al. 2009, 2011b) and *IGF2* (Murphy et al. 2011). In adult lung cancer patients, quantity and duration of active smoking as well as second-hand smoke is associated with increased DNA methylation of *p16* (Kim et al. 2001; Scesnaite et al. 2012), *MGMT*, and *DAPK* (Russo et al. 2005). Tobacco smoke is also associated with tumor cell DNA methylation changes in esophageal squamous cell carcinoma (Huang et al. 2011), significantly higher frequencies of abnormal DNA hypermethylation in prostate (Enokida et al. 2006) and gastric cancers tumor cells (Nan et al. 2005) and with a higher risk of CIMP + colorectal tumors (Limsui et al. 2010; Samowitz et al. 2006). Lastly, the *F2RL3* gene is hypomethylated in smokers and may mediate the detrimental impact of smoking on cardiovascular mortality, since hypomethylated *F2RL3* was found to be strongly associated with cardiovascular mortality among patients with stable coronary heart disease (Breitling et al. 2012).

#### Oxidative stress

Reactive oxygen species (ROS) are involved in numerous cellular processes including cellular redox alterations, immune response, signaling pathways, chromatin remodeling and gene expression (Sundar et al. 2010). ROS have the potential to influence epigenetic mechanisms (Baccarelli and Bollati 2009), and have been shown to inhibit binding of methyl-CpG binding protein 2, a critical epigenetic regulator that recruits cytosine methyl transferases and histone deacetylases to DNA (Valinluck et al. 2004). Numerous environmental exposures, including constituents of air pollution and tobacco smoke, can generate ROS and thus may potentially alter epigenetic processes through oxidative stress mechanisms.

#### Metals

Prenatal lead exposure is associated with decreased methylation of LINE1 and Alu in cord blood (Pilsner et al. 2009), and a similar pattern of LINE1 methylation was reported in an elderly cohort (Wright et al. 2010). Studies in humans have shown that arsenic exposure is associated with either global hypermethylation or hypomethylation in peripheral blood mononuclear cells (PBMCs) depending on dose (Majumdar et al. 2010), as well as DNA hypermethylation of several genes, including *CDKN2A* (Chanda et al. 2006), *RASSF1A* and PRSS3 (Marsit et al. 2006). Exposure to airborne particulates rich in lead, cadmium and chromium are associated with miRNA expression in peripheral blood (Bollati et al. 2010) and airborne levels of nickel and arsenic are positively correlated with both histone 3-lysine4 trimethylation (H3K4me3) and histone 3-lysine9 acetylation (H3K9ac) in blood leukocytes (Cantone et al. 2011). Occupational exposure to nickel is associated with increased H3K4me3 and decreased H3K9me2 in PBMCs (Arita et al. 2011). Lastly, cadmium can induce overexpression of the DNA methyltransferase genes *DNMT1* and *DNMT3a* in human embryo lung fibroblasts, and is associated with hypermethylation and silencing of the *MSH2, ERCC1, XRCC1* and *OGG1* genes in human bronchial epithelial cells (Jiang et al. 2008; Zhou et al. 2011).

#### Organic chemicals

Gas-station attendants and police officers occupationally exposed to low levels of benzene were found to have significantly lower LINE1 and Alu methylation, hypermethylation of *p15*, and hypomethylation of *MAGE*-*1* in blood (Bollati and Baccarelli 2010; Bollati et al. 2007).

### Genetic × epigenetic × environmental interactions

Most work investigating effects of environmental factors on epigenetic state has not considered the potential for genetic susceptibility to modify these associations. However, Salam et al. (2012) recently investigated contributions of both genetic and epigenetic variation in air pollution-mediated levels of fractional exhaled nitric oxide (feNO). Measurement of feNO provides an in vivo summary assessment of inducible nitric oxide synthase (iNOS) activity as well as airway inflammation. These investigators found interrelated effects of exposure to the air pollution constituents PM_2.5_, *NOS2A* promoter haplotypes, and methylation of the iNOS encoding gene *NOS2A* and *NOS2* promotor haplotypes on feNO level. These observations illustrate not only the feasibility of assessing interactions between epigenetic, genetic, and environmental factors, but also the importance of doing so in order to delineate complex biological relationships and identify susceptible subpopulations.

## Epigenetic effects in human disease

Conditions associated with improper parental contributions of imprinted genes are currently the clearest examples of human diseases related to epigenetic state. Even before genomic imprinting was described, experiments in which pronuclei were transplanted into enucleated eggs demonstrated that both maternal and paternal chromosomal contributions are required for normal development. Control conceptuses receiving one set (haploid genome) of maternal (egg-derived) and one set of paternal (sperm-derived) chromosomes could develop normally. However, abnormal development and early demise occurred in all conceptuses receiving either two maternal sets or two paternal sets of chromosomes (McGrath and Solter 1984), which developed into tissues with histologic features of dermoid cysts and hydatiform moles, respectively.

Model imprinting disorders such as Beckwith–Wiedemann, Angelman, Russell–Silver, and Prader–Willi syndromes are human conditions that can be caused by aberrant epigenetic state (Ferguson-Smith 2011). The specific features, early onset, and rarity of these disorders facilitated recognition of their relation to improper parental contributions of imprinted loci (e.g. two maternal or two paternal copies, rather than one maternal and on paternal copy), which can arise from uniparental disomy, more local epigenomic abnormalities (Tierling et al. 2011), or widely distributed changes caused by genetic defects in *trans*-acting epigenetic regulators (Begemann et al. 2011).

The role of epigenetics in most other conditions remains far less clear. However, since Barker proposed that there is sometimes a fetal basis for adult disease (Barker et al. 1993), epidemiologic research has implicated numerous environmental exposures during prenatal and early postnatal development as influencing risk of adult cardiovascular disease, obesity, type 2 diabetes, and other chronic conditions (Taylor and Poston 2007). Epigenetic processes have been proposed to explain lengthy time periods between exposure and disease onset: if exposures during early periods of epigenetic vulnerability influence epigenetic state, resulting epigenetic marks may be stably maintained in cell lineages to influence disease susceptibility years later. Waterland and Jirtle (2004) and Lillycrop (2011) recently discussed experimental data addressing the plausibility of such mechanisms. Human research described here provides correlative data. Associations between exposure history and epigenetic state are described in the previous section, while associations between epigenetic state and disease risk are outlined below, with a few studies reporting associations over the exposure—epigenetic state—disease continuum.

### Reproductive conditions

The possibility that epigenetic state can influence reproductive success was recognized once research in model organisms revealed that dramatic epigenetic reprogramming occurs during gametogenesis, fertilization, and development of the zygote, embryo and placenta (Feng et al. 2010). Ethical considerations have limited studies that directly measure epigenetic state to a small subset of relevant cell types and developmental stages. Here we review selected data addressing the hypothesis that epigenetic phenomena may influence risk of infertility, disorders of placental development and function, and birth weight.

Human infertility research has assessed DNA methylation profiles in DNA isolated from spermatozoa, fully differentiated male germline cells that are amenable to noninvasive collection. Although neither measures of DNA methylation nor outcomes have been standardized across published studies, most report variation in epigenetic state of sperm DNA to be associated with undesirable outcomes (Carrell and Hammoud 2010). Early studies employing methylated cytosine immunostain—a genome-wide measure without sequence specificity—revealed little difference between sperm DNA methylation and sperm quality (Benchaib et al. 2003) or fertilization rate and embryo quality following in vitro fertilization (IVF) (Benchaib et al. 2005). However, all subsequent studies assaying specific sequence elements (Pacheco et al. 2011, reviewed in Cortessis et al. 2011; van Montfoort et al. 2012) reported abnormal methylation of one or more imprinted genes in DNA from poor quality sperm, as predicted if sperm production defects involve deregulated imprinting (Filipponi and Feil 2009). Three studies assaying repeated elements reported no association between sperm quality and methylation of Alu (Kobayashi et al. 2007) or LINE1 (Houshdaran et al. 2007; Marques et al. 2008), while one reported elevated Alu methylation in sperm used in assisted reproductive technologies (ART) leading to pregnancy and live birth (El Hajj et al. 2011) and another that poor sperm quality was associated with hypermethylation of the repetitive element SAT2CHRM1 (Houshdaran et al. 2007). Studies interrogating CpG islands not associated with recognized imprints reported methylation at numerous sites to be associated with sperm quality, implicating widespread disruption of DNA methylation in abnormal spermatogenesis (Houshdaran et al. 2007; Pacheco et al. 2011). This avenue of research is expanding to address additional epigenetic marks of specific relevance to the male germline (Carrell and Hammoud 2010; van Montfoort et al. 2012).

Early stages of germ cell development are reportedly identical in both sexes, so early disruptions of epigenetic state could in theory impair fertility of both males and females. However, direct epigenetic study of female gametes is not regarded as practicable, because very few oocytes are produced, and oocyte retrieval procedures may alter epigenetic state (Fortier et al. 2008; Li et al. 2011; Sato et al. 2007).

Epigenetic state has also been implicated in infertility by a distinct form of data, excess occurrence of model imprinting disorders among children conceived by ART (van Montfoort et al. 2012). A recent meta-analysis found elevated occurrence of imprinting disorders of both maternal origin (Angelman syndrome, Beckwith–Wiedemann syndrome) and paternal origin (Prader–Willi syndrome) (Cortessis 2009). Because only sub-fertile individuals use ART, these results could indicate a tendency for sub-fertile adults to produce gametes with inappropriately set imprint marks. However, whether underlying molecular defects of these children are epigenetic or genetic was not reported, and even if they were epigenetic, alternate explanations are credible. Couples undergoing ART are dissimilar to the general population with respect to age and income and thus numerous unmeasured factors that could be postulated to influence epigenetic state. Moreover, ART procedures bypass various functions that accompany unassisted conception. These include the need for spermatozoa to traverse the female reproductive tract and compete to penetrate the oocyte, which may serve to select for fertilization sperm with normal epigenetic state. Finally, effects of ART themselves have been postulated, since some imprinting disorders share overgrowth features of the veterinary condition large offspring syndrome, which appears to be inducible by embryo culture (Sinclair et al. 2000). However, data on epigenetic state of human oocytes and embryos are extremely limited (van Montfoort et al. 2012).

Fetal survival, development and growth depend upon the placenta. Arising from the relatively hypomethylated TE, cells of the developing placenta undergo extensive de novo DNA methylation (Serman et al. 2007). Epigenetic regulation of placental function changes during gestation (reviewed in Nelissen et al. 2011), accompanied by a dynamic process of loss of imprinting postulated to have an important role in placental maturation and function (Pozharny et al. 2010). Unidentified placental functions are postulated to rely on imprinting, because imprinted genes are abundantly expressed in the placenta, compared with other organs, and imprinting arose during mammalian evolution (Nelissen et al. 2011). Disturbed placental function may cause intra-uterine growth restriction (IUGR), a predictor of poor health in the neonatal period and childhood, which may also predispose to diseases of adulthood. Two small studies examining mRNA transcript levels in placentas of IUGR and normal birthweight infants reported differential expression of some placentally imprinted genes (Diplas et al. 2009; McMinn et al. 2006); a third reported lower IGF2 and loss of *IGF2* imprints (Koukoura et al. 2011a) as well as higher *H19* transcript levels and increased *H19* expression (Koukoura et al. 2011b) in growth-restricted placentas. Differential levels of transcripts encoded by individual imprinted loci were also reported in IUGR placentas (Apostolidou et al. 2007) and small for gestational age infants (Guo et al. 2008).

Infants conceived by ART have been shown consistently to have lower birthweight than other infants (Andersen et al. 2008). A recent meta-analysis revealed that in published studies of singleton births controlled for effects of maternal age, low birthweight was more common among those conceived by IVF (McDonald et al. 2009). In the same review, IVF conception was also associated with premature birth, which is highly associated with low birthweight. Similar analyses indicate that low birthweight and preterm birth are also more common among twins conceived by IVF compared with naturally conceived twins (McDonald et al. 2010). Addressing ART procedures more generally, recently published surveillance data on over 16,000 births revealed elevated occurrence of low birthweight, small-for-gestational age, and preterm birth among singleton but not twin infants conceived following ART (D’Angelo et al. 2011).

It is presently not clear whether low birthweight following ART reflects epigenetic state of gametes inherited from sub-fertile parents, effects of ART, or other unrecognized factors. Additional insight may accrue from future research addressing possible reasons that ART conception is associated with model imprinting disorders and low birthweight, as well as recently initiated investigation of any relationship between epigenetic state and these outcomes in naturally conceived neonates (Lee et al. 2012).

### Asthma

Emerging evidence suggests that the epigenetic state may contribute to asthma pathogenesis. Asthma is an IgE-mediated type I hypersensitivity reaction involving skewed programming of naïve CD4+ T lymphocytes toward a Th2 lineage (Durham et al. 2011). Shift toward a Th2 phenotype may result from chromatin remodeling brought about by multiple, coordinated epigenetic changes on genes regulating Th differentiation (Ho 2010). These include loss of DNA methylation and gain of H3K9ac and H3L4me2 in *IL*-*4* and *IFN*-*γ*, genes that encode key cytokines involved in T cell lineage development (Fields et al. 2002; Jones and Chen 2006; Kwon et al. 2008; White et al. 2002). DNA methylation of the *Foxp3, ACSL3*, and *ARG2* loci are associated with impaired regulatory T-cell function (Nadeau et al. 2010), increased asthma morbidity (Nadeau et al. 2010; Perera et al. 2009), and exhaled nitric oxide production, respectively (Breton et al. 2011a). Histone deacetylases (HDACs) have also been shown to maintain pre-established Th1-like and Th2-like immunity in human T cells; inhibition of HDAC can shift the Th1:Th2 ratio, skewing response toward Th2-like phenotype (Su et al. 2008). Additional evidence implicates miRNAs in asthma pathogenesis (Pauley and Chan 2008). MiR-126 expression-suppressed asthmatic phenotype (Mattes et al. 2009) and differentially expressed miRNAs including miR-21, -103, -155, -146a, and -204 were identified in response to innate and adaptive immune stimuli (Liu et al. 2009; Nana-Sinkam et al. 2009).

### Metabolic diseases

Functional epigenetic changes have been observed in obesity (Campion et al. 2009), diabetes (Reddy and Natarajan 2011), cardiovascular disease (Wierda et al. 2010), and the constellation of abnormalities known as metabolic syndrome (Bruce and Hanson 2010). For example, adverse vascular effects of hyperglycemia can continue for several years following sufficient glycemic control, a phenomenon called metabolic memory (Siebel et al. 2010), and there is evidence from animal studies that this effect is associated with epigenetic changes in the promoter of the NFκβ subunit of the *p65* gene in aortic endothelial cells (El-Osta et al. 2008) and increased acetylation at H3K9/K14 in a number of genes involved in endothelial dysfunction and chronic inflammation (Pirola et al. 2011). In women, promoter-region methylation of *GNSAS* and *INSIGF* in leucocytes was reportedly associated with an increased incidence of myocardial infarction (Talens et al. 2011), suggesting that epigenetic modification may be clinically relevant. Although various histone modifications have been demonstrated in vitro in relation to complications of hyperglycemia (Wierda et al. 2010), permanence of individual changes has not been established.

### Neurological disorders

Historically, the field of epigenetics has focused on elucidating mechanisms for maintaining DNA methylation in dividing cells. However, recent work has discovered dynamic DNA methylation changes in non-dividing cells including neurons (reviewed in Mill et al. 2008), motivating studies of effects of environmental exposures on epigenetic marks in relation to neurological disorders.

Alzheimer’s disease, schizophrenia, and autism spectrum disorders show a variety of epigenetic anomalies (Akbarian 2010; Mill et al. 2008; Nguyen et al. 2010b; Schroeder et al. 2011). An epigenetic mechanism has been proposed to explain the association between famine during the prenatal period and schizophrenia risk (Lumey et al. 2011), as well as associations between expression of imprinted genes and both autism and schizophrenia (Badcock and Crespi 2008). Recent studies, moreover, report associations of both conditions with DNA methylation in additional, non-imprinted genes (Dempster et al. 2011; Mill et al. 2008; Nagarajan et al. 2006; Schroeder et al. 2011).

### Cancer

Abnormal DNA methylation, histone code modifications and miRNA changes have all been demonstrated in human cancer cells as reviewed elsewhere (Kanwal and Gupta 2011; Lovat et al. 2011; Sharma et al. 2010). In malignant tumors, gradual genome-wide DNA hypomethylation is observed, concurrent with hypermethylation at normally unmethylated CpG islands (Baylin and Jones 2011; Feinberg 2007). Although a direct association between hyper- and hypo-methylation was not initially recognized (Bariol et al. 2003; Iacopetta et al. 2007), recent work suggests that the two phenomena may well be linked, and are confined to similar compartments of the genome (Berman et al. 2012). Although the targeting of abnormal hypermethylation in gene promoter regions is not completely understood, its association with occupancy by polycomb complex proteins (Ohm et al. 2007; Schlesinger et al. 2007; Widschwendter et al. 2007), which transiently repress genes involved in differentiation, suggests that such methylation may be associated with either de-differentiation or a failure of tissue stem cells to fully differentiate.

Although most cancers show aberrant methylation of some gene promoter regions, a subset of tumors is characterized by CIMP, a phenotype involving concurrent methylation of multiple gene promoter regions (Toyota et al. 1999; Weisenberger et al. 2006). This phenotype is best described in colorectal cancer, for which a carefully vetted set of markers (Weisenberger et al. 2006) allows epidemiologic studies of risk factor differences between CIMP and non-CIMP tumors (reviewed in Curtin et al. 2011). The association between smoking and colorectal cancer was reported to be limited to those with the CIMP phenotype (Limsui et al. 2010; Samowitz et al. 2006). Additionally, integration of the hepatitis B virus (HBV) into hepatic cell DNA has been associated with increased DNA hypermethylation in hepatocytes (Herceg and Paliwal 2011) or alternatively simply reflect a consequence of increased cell turnover. Chronic inflammation may also modify epigenetic marks on both histones and DNA (Bayarsaihan 2011). Other environmental factors have also been associated with aberrant DNA methylation in some human studies (reviewed in Lim and Song 2012) but further study of additional populations is needed.

The role of folates and other one-carbon nutrients, which are critical in the provision of methyl groups, has been assessed in many epidemiologic studies of abnormalities in methylation in CRC, with largely disappointing results (Curtin et al. 2011). However, folates and polymorphisms in genes of the folate pathway have been shown to modify global DNA methylation in circulating lymphocytes (Lim and Song 2012), although the relationship between such changes and changes in target tissues is not known. Additionally, the risk of various childhood malignancies may be significantly decreased in children exposed in utero to higher folate and other one-carbon nutrients, mainly via multivitamin use (reviewed in Ciappio et al. 2011). Although a direct correlation between maternal one-carbon nutrient levels and aberrant DNA methylation in either childhood or adult tumors has not been demonstrated, these studies provide a basis for future research into the relationship between prenatal exposure to methylation-associated nutrients and the abnormal methylation events so common in carcinogenesis.

## Transgenerational phenomena

In theory, epigenetic marks in somatic cell lineages could be transmitted to daughter cells over the lifespan of an exposed individual, while marks established in germ cell lineage members could be transmitted to subsequent generations. Several examples of apparent transmission of an environmental insult across generations are described below. However, whether epigenetic mechanisms mediate these phenomena remains highly controversial (Bollati and Baccarelli 2010). Convincing evidence that an epigenetic mechanism is responsible for transgenerational effects requires determination of the phenotype in at least three generations following exposure. Taking as F0 the exposed pregnant mother, observations of F1 through F3 generations are required to exclude the possibility of direct effects of the exposure both on somatic cells of the F1 generation or on germ cells progenitors from which the F2 generation will arise, since both are present during the exposed pregnancy (Perera and Herbstman 2011). It is also possible that selection of pregnancies for viability could influence the distribution of genes that influence epigenetic marks.

### Experimental animals

The best-documented example of transgenerational specification of a phenotype through DNA methylation is the determination of coat color and other traits (obesity, diabetes, and tumors) in agouti mice. Dams carrying the meta-stable A^vy^ epiallele (who themselves display the agouti coat pattern) are more likely to produce agouti offspring, while dams of the same strain without the A^vy^ epiallele (who have yellow coats) are more likely to produce yellow offspring. A continuous gradient in coat color is related to the penetrance of the A^vy^ epiallele (Morgan et al. 1999; Waterland and Jirtle 2003) determined in part by methyl donors in the dam’s diet. The phytoestrogen genistein can also change the epigenetic state of this allele for several subsequent generations (Cropley et al. 2006; Dolinoy et al. 2007a; Lillycrop et al. 2005). While these observations convincingly argue for epigenetic determination of the agouti phenotype, the meta-stable A^vy^ epiallele arose from insertion of a retrotransposon rather than from conventional environmental exposure.

In a separate model, experimental exposure of pregnant rats to endocrine-disrupting agents (vinclozolin, bisphenol A) produced changes in male infertility, body weight, and cancer risk in up to four subsequent generations (Anway et al. 2005; Anway and Skinner 2008). However, whether specific epigenetic marks determine this phenotype across these generations has not been established.

### Human studies

#### Famine

Pembrey et al. (2006) described an association between paternal grandfather’s exposure to a famine in northern Sweden and mortality in their grandsons, whereas paternal grandmother’s food supply was associated instead with mortality in their granddaughters. Lumey (1992) reported effects on birth weight up to two generations following the Dutch famine in World War II.

#### Tobacco smoke

The Southern California Children’s Health Study has demonstrated an effect of grandmaternal smoking while pregnant on the asthma risk of their grandchildren, after controlling for maternal smoking and environmental tobacco smoke exposure (Li et al. 2005). Pembrey et al. (2006) also described effects of pre-adolescent paternal smoking on BMI in sons but not daughters. Although any of these phenomena could be due to epigenetic mechanisms, mutation to the DNA itself would be a plausible alternative, as has been shown for mainstream tobacco smoke (Yauk et al. 2007).

#### Diethylstilbestrol (DES)

Decades ago, human males exposed in utero to DES were found to have excess occurrence of urogenital malformations, including hypospadias (Henderson et al. 1976; Vessey et al. 1983; Whitehead and Leiter 1981), and elevated prevalence of hypospadias was subsequently reported among unexposed boys whose mothers had been exposed in utero to DES (Brouwers et al. 2006; Klip et al. 2002; Palmer et al. 2005; Pons et al. 2005). More recently, in a study addressing potential competing effects of measured environmental and genotypic factors, a notable excess of hypospadias was reported among unexposed grandsons of women who had been exposed in utero to DES (Kalfa et al. 2011). One postulated mechanism for these observations is that epigenetic changes in the androgen receptor gene are induced in primordial germ cells of DES-exposed female fetuses at the time that the reproductive system is forming, and subsequently transmitted across generations to affected sons and grandsons (Kalfa et al. 2011). To our knowledge, this mechanism has not been examined at the molecular level.

## Methodological challenges

### Exposure assessment

The long induction periods between exposure and onset of many diseases that epigenetic mechanisms are proposed to mediate present notable challenges to accurate assessment of environmental exposures. Most exposures can change over time, complicating both accurate recall and assessment by short-lived biomarkers. Distinguishing exposures of limited duration or periods of specific relevance (e.g., in utero) from chronic exposures may lend useful specificity for addressing some research questions. Persistent biomarkers (e.g., bone lead levels), when available, are promising if the specific period of exposure is not crucial. Exposure assessed prospectively and tightly linked to proposed periods of vulnerability of the epigenome (e.g., periods of placental invasion or sex specification in utero) would be ideal. However, this rarely occurs in epidemiologic settings with sufficient power to detect most disease associations. However, estimating exposure–epigenetic state associations may be more feasible.

Exposures of limited duration corresponding to disasters (e.g., famine), availability of regulated or withdrawn compounds (e.g., DES), or therapeutic indications of limited duration (e.g., ART) can sometimes be retrospectively assessed with reasonable accuracy. Cohorts based on such exposures, although rare and often small, may be especially valuable in efforts to elucidate transgenerational processes.

Since assessment of covariates may be equally challenging, there is considerable potential for confounding of exposure–epigenetic state or exposure–disease associations by factors associated with exposure, except in rare circumstances when exposure can be randomly assigned to study participants.

### Laboratory technologies

Traditional molecular biological methods of analyzing gene loci often involve amplification through cloning, whole-genome amplification (WGA), or polymerase chain reaction (PCR). All of these techniques erase any existing marks in extracted DNA or chromatin. Therefore, methods have been developed to alter the DNA using methods influenced by the epigenetic state prior to amplification. The three main methods employed are affinity enrichment, restriction digestion, and chemical conversion by sodium bisulfite (Laird 2010). Affinity enrichment is appropriate for most epigenetic marks, generally relying on the use of an antibody specific for the mark of interest to enrich for DNA sequences containing the relevant mark by chromatin immunoprecipitation (ChIP). Affinity enrichment can also be used for DNA methylation, but restriction digestion with methylation-resistant and sensitive restriction enzymes has also seen widespread use. Treatment of denatured DNA with sodium bisulfite can convert unmethylated cytosines to uracil, while leaving methylated cytosines intact, thus converting epigenetic information into a genetic difference. As microarray technology became more widespread in the past decade, restriction enzyme and affinity-based methods became popular. With the development of commercial array platforms and the surging use of next-generation sequencing, bisulfite-based methods are now the most widely used methodology, and are well-suited for the types of biospecimens collected in epidemiologic studies.

### Biospecimens

The requirement of relatively large amounts of intact chromatin for ChIP-chip or ChIP-seq has limited the study of histone modifications and other protein-associated epigenetic marks to biospecimens with large amounts of material with intact cells. The vast majority of studies on ChIP have used cultured cell lines. More recently, techniques to analyze small quantities of fresh tissue have been explored (Adli and Bernstein 2011). However, none of the existing techniques for analysis of histone modifications are ready for use on the types of samples that have typically been collected in population-based studies, such as blood, buccal swabs, newborn dried blood spots, or formalin-fixed and paraffin-embedded (FFPE) samples. Therefore, most studies have relied almost exclusively on the analysis of DNA methylation, which can be performed on small quantities of extracted DNA. Even heavily cross-linked and degraded DNA obtained from FFPE samples can be interrogated for the distribution of methylation marks.

Investigation of epigenetic mechanisms requires consideration of specific type or types of cells to be studied, since normal epigenetic state varies between cell types (Wu et al. 2011). For many diseases and exposures, the relevant affected cells or tissue may not be readily accessible or even known. The cell types of greatest relevance are unlikely to be represented in extant biorepositories assembled for genetic research, for which most nucleated cells suffice, making minimally invasive collection a primary consideration. Proxy tissue may in special circumstances be useful following deliberate validation (Talens et al. 2010). Where cells of greatest relevance are collected several concerns remain: there may be ethical barriers to obtaining relevant cells from unaffected control individuals or even from cases; cells may not be sufficiently abundant or amenable to separation from surrounding tissue; and if disease processes alter proportions of normal cells spurious associations between epigenetic state and disease may occur on the basis of normal epigenetic profiles alone (Wu et al. 2011).

### Statistical methods

Statisticians have developed various methods for disentangling imprinting, parent-of-origin, and maternal–fetal interaction effects in family data (Ainsworth et al. 2011). These generally entail extensions of the transmission-disequilibrium test, which seeks departures in genotype distributions among affected offspring from expected distributions given their parents’ genotypes. The standard test ignores differences between the two parents, so that aA × aa and aa × aA mating types would be considered interchangeable. However, by adding additional terms to the conditional likelihood to distinguish between maternal and paternal alleles, as well as interactions between maternal and offspring genotypes and by incorporating grandmaternal genotypes, it may be possible to separate these phenomena. Rather than the standard likelihood framework for family-based association studies, Weinberg and colleagues (Vermeulen et al. 2009) have developed a flexible log-linear modeling framework based on the joint distribution of mating types and offspring genotypes and extensions to include grandparental genotypes. One particularly important development is the “pent” design (Mitchell and Weinberg 2005) for studying maternal–fetal interactions, entailing genotypes of a mother, her offspring, her spouse, and her parents; in this design, paternal grandparents are not needed, as their mating type can be imputed. However, none of these methods directly incorporate measured epigenetic states.

Development of statistical methods for analysis of DNA methylation, the most frequently measured epigenetic mark, has been dispersed across applications using different technologies. Although a large body of work is starting to accumulate for microarrays, there are still three major platforms in use, each with their own statistical analysis methods (see Siegmund 2011 for a review). Cross-platform adaptation of some methods (e.g., subset quantile normalization Aryee et al. 2011) is an area of current investigation.

Most methods for biological analyses focus on either searching for new disease subtypes through cluster analysis (Clifford et al. 2011; Siegmund et al. 2004), or looking for areas of differential DNA methylation between samples under different conditions (Jaffe et al. 2012a; Langevin et al. 2011). Sometimes differential DNA methylation is defined by differences in variation, rather than a shift in the mean (Jaffe et al. 2012a).

There has been relatively little emphasis on modeling epigenetic state as an intermediate variable. In one such example, however, Siegmund et al. (2006) used a finite mixture model to estimate the association between exposure and latent disease subtype measured by DNA methylation profiles and compared these results with a simpler two-phase approach, first clustering the DNA methylation data and then relating these clusters to exposure using logistic regression.

Several authors (Furrow et al. 2011; Slatkin 2009; Tal et al. 2010) have developed models of epigenetic inheritance that allow epigenetic state to be reset between generations, as well as environmental induction and environment-sensitive modifications. They have shown that variation in either the environmental or the epigenetic state could produce high heritability, comparable to the heritability expected from purely genetic effects. These papers, however, are focused more on theoretical assessment of how much of the “missing heritability” could be due to epigenetics rather than providing methods for analysis of epidemiologic data. We therefore briefly outline a general framework that could be used for this latter purpose. Details and supporting simulation studies will be described completely elsewhere.

One approach could be to treat an individual’s epigenetic state as a time-dependent latent process, μ_*i*_(*t*), which is only partially observed at particular time points *t*_*k*_ by flawed assays *M*_*ik*_. Using standard measurement error modeling approaches, one might then treat the latent process as being determined by one’s constitutional genotype *G*_*i*_ and exposure history *E*_*i*_(*t*), through a linear longitudinal random-effects model, with the latent process in turn being a risk factor for disease state *Y*_*i*_(*t*), through a standard survival analysis model (see, for example, Elashoff et al. 2007) for similar latent process methods applied to longitudinal data on CD4 cell counts in relation to AIDS incidence). To address the high-dimensional nature of epigenetic data, one could use the kinds of cluster-analysis techniques described by Siegmund et al. (2006), treating the cluster rather than individual epigenetic marks, as a latent risk factor for disease (see Molitor et al. 2003 for an example of similar latent cluster methods applied to haplotype associations).

**Fig. 2:**
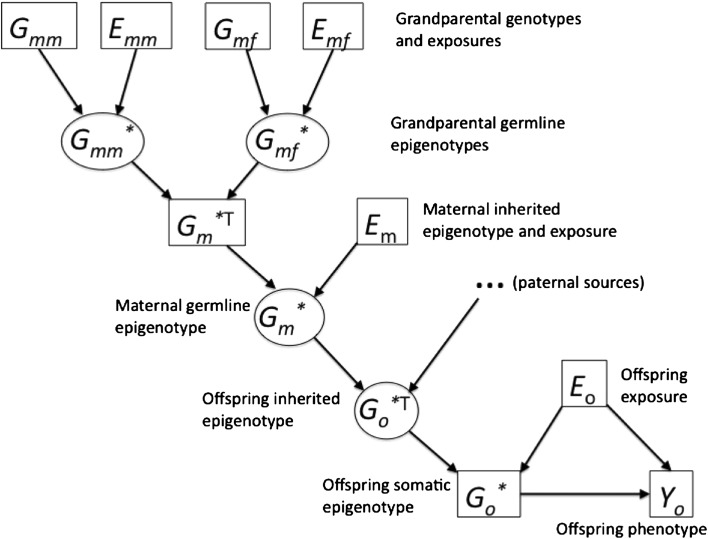
Schematic representation of the model for transgenerational effects

To address transgenerational phenomena, we propose an extension of this approach illustrated in Fig. 2. In this model, grandparental genetic and environmental effects (*G*_*mm*_ *,E*_*mm*_ etc.) are transmitted to the parents via transmitted epigenotypes (*G*_mm_* etc.), which are modified by the parents’ environments and in turn transmitted to the offspring generation to determine their phenotypes, in combination with their own exposures at the relevant developmental stage.

### Extending to genome-wide scale

Much of the early work on the role of epigenetics in disease has focused either on global methylation, differentially methylated regions associated with several imprinted loci, or promoter regions of selected candidate genes. Now, facilitated by the advent of high-density methylation arrays like the Illumina Infinium Beadarray that can interrogate up to 480K CpG sites, the kinds of agnostic genome-wide scans that have become popular for investigating germline genetic variation [genome-wide association scans (GWAS)] are likely to become widely used for epigenetics as well. There are two forms such studies could take. The first is a genome-wide scan for epigenetic variation in relation to disease risk—what has recently been called an epigenome-wide association scan (EWAS) (Rakyan et al. 2011) (this should not to be confused with the use of the same acronym for exposure-wide association scans, Patel et al. 2010). For example, in an agnostic scan of 92 head and neck cancers and 92 controls, 6 CpG loci were identified (Langevin et al. 2012). Here, the focus was on finding biomarkers that might be useful for early detection rather than for etiologic inference. Case–control designs are commonly used in GWAS studies, wherein measured biomarkers of interest are genotypes that do not change over time and are presumed to be identical in all nucleated cells of each individual. EWAS for etiology are far more challenging for two fundamental reasons. First, use of retrospective case–control designs introduces the possibility of “reverse causation,” wherein the disease or its treatment causes epigenetic changes rather than the reverse. Second, tissue and cell type specificity of epigenetic marks measured in EWAS pose the issues of sample collection and interpretation described in the previous section.

A different example, provided by an EWAS of the effect of smoking on methylation (Breitling et al. 2011), which found lower methylation at a CpG site in *F2RL3* in smokers than nonsmokers, avoids the problem of reverse causation, but not of tissue specificity.

The second approach to agnostic genome-wide epigenetic research uses methylation quantitative trait loci (mQTLs). These seek genome-wide SNP variation associated with genome-wide epigenetic state (Bell et al. 2011a) in a manner similar to expression QTL (eQTL) studies. Studies of mQTLs offer the potential to detect both *cis*-associations (SNPs in or near a gene that are associated with epigenetic state of the *same* gene) and *trans*-associations (associations with epigenetic state of genes located at some distance from the SNP or on separate chromosomes). Although the vast majority of associations in HapMap cell lines studied by Bell et al. were of the *cis* type, they found a *trans* association of a SNP in the *DIP2B* gene (thought to be involved in DNA methylation) with the first principal component of genome-wide methylation.

EWASs pose novel statistical challenges for peak detection that differ from other applications of this techniques (such as for ChIPseq data), as summarized by Jaffe et al. (2012b). Not only a larger number of individuals studied, but the correlation structure from adjacent probes on the microarray is more complex, peaks are expected to be more variable in size and shape and in certain applications more numerous (requiring different approaches for multiple comparisons) and there can be more serious measurement error problems. Jaffe et al. propose a novel “bump hunting” technique to address such problems. Another challenge concerns the problem of reverse causation mentioned above. In studies of the association of biomarkers with disease, the technique of Mendelian randomization (Davey Smith and Ebrahim 2004) has received much attention as a way of overcoming reverse causation and uncontrolled confounding by using a gene as an instrumental variable (Greenland 2000) to assess the causal effect of the biomarker on disease risk. Relton and Davey Smith (2012) have proposed a novel two-step extension of this idea for methylation studies, using two genes as instrumental variables, one to estimate the exposure–methylation association, the other to estimate the methylation–disease association.

## Epidemiological opportunities

Despite the wealth of research on the role of epigenetics in disease, there has been much less activity on the environmental determinants of epigenetic changes and their role of epigenetic state in mediating exposure–response relationships, either within individuals or across generations. Rakyan et al. (2011) provide an excellent review of some of the design issues in epigenetic epidemiology. Here we focus on some opportunities for further epidemiologic study in this area.

### Case–control and cohort studies

Studies of the genetic determinants of disease, whether based on candidate site hypotheses, pathway-based hypotheses, or GWAS technology, have utilized cross-sectional studies in what is conceptually a case–control approach, since the “exposures” in question were genotypes presumed to be immutable. Analogous studies of disease outcomes in light of epigenetic alterations, however, are fundamentally flawed, not only because earlier causal events could always be responsible for observed epigenetic change, but also because any stage in the pathophysiology of the disease and/or any treatment prior to sample collection could itself generate epigenetic change. De novo cohort studies of the genetic determinants of disease require biosamples at the outset, are slow to yield results, and often prohibitively expensive, because the rare outcomes of greatest interest can only be assessed if the genomic subsets are very large. Cohort studies of the causation of endpoint phenotypes or even intermediary risk factors by epigenetic characteristics are likely to be just as difficult. Moreover, observational studies of human epigenetic events as usually conducted are confounded by age, genomic characteristics, and the earlier environmental milieu, including the prepartum component.

One can, however, study epigenetic alterations as outcomes, and by so doing, come to understand their role as intermediary agents between disease outcomes and the toxic agents known to cause them. Cross-sectional studies of cells from exposed and unexposed individuals are interpretable under the assumption that the epigenetic alterations are unlikely to have produced the behavior that led to the toxic exposure. Over the long term, comparison between the epigenetic experiences of exposed and unexposed cohorts will require repeated epigenetic characterization of persons with particular toxic exposures or particular characteristics, such as jobs that stand as surrogate measures of such exposure.

### Twin studies

In contrast to observational studies of singletons, twin studies can provide several advantages for studies of epigenetics (Bell and Saffery 2012). Monozygotic twins are in effect matched on age, complete genome, and childhood environment and cultural milieu. By contrast, even monozygotic twins can be considered only partly matched on intrauterine environment. Because epigenetic events occur at various points throughout gestation, epigenetic state at birth is probably influenced by chorionicity and possibly as well by characteristics of the amnion and the anatomy of implantation.

Differences between the global and gene-specific epigenetic states of adult identical twins have been described in numerous studies (Bell et al. 2012; Bocklandt et al. 2011; Boks et al. 2009; Fraga et al. 2005; Heijmans et al. 2007), suggesting that epigenetic state changes with age, or more accurately, with cohort, since these were all different people. Methylation state at some CpG sites is highly predictive, as shown in a recent study that identified numerous methyl marks in DNA isolated from saliva to be highly associated with age. In a validation set of study participants, methylation state at only two of the identified CpG sites explained 73 % of the variance in age over a 5-decade range (Bocklandt et al. 2011). Such epigenetic marks may prove useful not only in understanding biological process associated with aging, but also as tools for classifying participants in research into biologically based age groups. For example, individuals with large differences between epigenetically predicted age and chronological age might be considered to have unusual physiologic state or bio-age.

However, unless age-specific epigenetic states are demonstrated in the *same* twins studied across different ages, as has been done in singletons (Bjornsson et al. 2008), apparent effects of age on epigenetic state may be confounded by birth cohort-specific differences in exposure history. In a study of identical twin children observed at both ages 5 and 10, prevalence of DNA methylation at selected promoter sites changed measurably over time (Wong et al. 2010), as did prevalence of more extreme skewing of X chromosome inactivation within some female pairs (Wong et al. 2011). Observed concordance between co-twins of changes in such measures provide information regarding factors that may influence epigenetic changes that is not available from singletons. For example, observing little concordance of within-pair changes in X chromosome inactivation, Wong et al. (2011) concluded that alterations in these patterns between 5 and 10 years of age are unlikely to be influenced by factors shared by twins. Establishment of cohorts to be periodically monitored for epigenetic changes should provide further documentation of age-specific changes (Saffery et al. 2011).

Identical twins discordant for both epigenetic state and a putatively toxic exposure or adverse circumstance can therefore be used to tentatively identify factors that cause or facilitate epigenetic change. For example, in a study comparing members of monozygotic dichorionic twin pairs, DNA from placental tissue of smaller co-twins was found to have lower prevalence of promoter DNA methylation at the human endogenous retroviral family W Env(C7) member 1 gene (*HERVWE1*). Moreover, mRNA and protein encoded by *HERVWE1* was more highly expressed in placental tissue of smaller co-twins (Gao et al. 2012).

Studies identifying differences in epigenetic state between identical twin pairs discordant for a trait or disease phenotype can raise provocative etiologic questions. However, as in case–control studies of phenotypic outcomes in general, these studies must be interpreted with caution, particularly if the chronology of relevant exposures, occurrence of epigenetic changes, and onset of pathophysiologic processes is unclear, if the variable locus is not known to function in the pathogenic pathway, or if the disease pathology is not manifested in the tissue in which epigenetic state is measured. Such provocative questions have been raised by studies of trait-discordant monozygotic twins in relation to differential X-inactivation in the context of bipolar disorder (Rosa et al. 2008) and primary biliary cirrhosis (Mitchell et al. 2011), and differential DNA methylation at specific loci in the context of a caudal duplication anomaly (Oates et al. 2006), bipolar disorder (Dempster et al. 2011; Kuratomi et al. 2008), schizophrenia (Dempster et al. 2011), and BRCA1-related malignancy (Galetzka et al. 2012). Global changes in methylation, to differing degrees, have been reported in identical twins discordant for psychometric evidence of aberrant personality (Kaminsky et al. 2008), Alzheimer’s disease (Mastroeni et al. 2009), systemic lupus erythematosus (Javierre et al. 2010), autism (Nguyen et al. 2010a), and inflammatory bowel disease (Harris et al. 2012). A comprehensive global search for differences between (only three pairs of) twins discordant for multiple sclerosis was unsuccessful, possibly because peripheral white blood cells were used (Baranzini et al. 2010). Differences in gene expression between trait-discordant identical twins, presumably reflecting epigenetic differences, have been observed for rheumatoid arthritis (Haas et al. 2006), bipolar disorder (Matigian et al. 2007), schizophrenia (Kakiuchi et al. 2008), type 1 diabetes (Beyan et al. 2010), diverse systemic autoimmune conditions (O’Hanlon et al. 2011), and psoriasis (Gervin et al. 2012).

Twins have also been used to suggest the existence of heritable determinants of epigenetic change. The traditional design is based on the comparison of proband-wise concordance between monozygotic twins with that between like-sex dizygotic twins, separating the variance attributable to the environment from that attributable to the additional half of the genome held in common between monozygotic twins. Reservations about conclusions from such studies are based on two considerations. The first concerns the assumption that the sharing of the monozygotic twins’ environment is identical to that of dizygotic twins—an assumption necessary for heritability to be estimable from twin studies. This assumption is suspect in adulthood, since dizygotic twins tend to diverge geographically and socially to a greater extent than do monozygotic twins, and since any monozygotic twin is more likely than a dizygotic twin to perceive and adopt a behavior seen to be advantageous to the co-twin. This is compounded by the second concern, namely the inevitable variability in the twin concordance of a phenotype that is also determined by a geographically, chronologically, or socially variable environmental exposure. Thus, the reliability of any such estimate is highest for phenotypes that are not confounded by age, time, and place, and especially for those of young children that have no behavioral determinants.

Moreover, when comparisons of twin concordance are used to estimate the heritability of an epigenetic trait, necessary caveats are more numerous. Heritability of epigenetic traits is clearly tissue specific, and likely to be locus specific as well. Because of the influence of the intrauterine environment and the fact that they exchange blood cells, monochorionic identical twins cannot be considered comparable to dizygotic twins, and comparisons should be based only on dichorionic monozygotic pairs. Irrespective of chorionicity, identical twins begin life as a single zygote, restricting the number of early cell-days at risk of independent epigenetic change relative to the separate zygotes of dizygotic twins.

Initial attempts at demonstrating heritability at specific loci by these methods have had some limited success (Boks et al. 2009; Breton et al. 2011c; Coolen et al. 2011; Heijmans et al. 2007; Schneider et al. 2010), but investigations of larger portions of the epigenome in specific tissues have shown only a marginal increase in epigenetic similarity between the members of monozygotic twin pairs, compared with dizygotic twin pairs (Gervin et al. 2011; Ollikainen et al. 2010).

### Multigenerational studies

In the remainder of this section, we discuss a few of the unique exposure settings that might be useful for exploring the role of epigenetics in apparent transgenerational exposure phenomena.

#### Famine cohorts

Testis cancer incidence has been increasing for decades, and age–period–cohort analyses consistently demonstrate that birth year is an important determinant of testicular cancer risk. Based on these findings, exposure to unidentified environmental causes is postulated to be increasing, with observed birth cohort effects attributed to early action of such exposures. The singular exception to this pattern is a dip in incidence among men born in Europe during World War II (Moller et al. 1995). Postulated epigenetic mechanisms related to energy intake in the in utero and perinatal periods warrant investigation among individuals gestated during famine and their descendents.

#### Child health and development studies

The California Child Health and Development Studies (Cohn 2011; van den Berg et al. 1988) were launched between 1959 and 1967 with the enrollment of a cohort of about 20,000 pregnancies, with follow-up of the parents and offspring through the present day. Biological specimens were provided by both parents and the child at the time of enrollment, along with limited questionnaire information about grandparental exposures, and longitudinal samples have also been obtained. As these children are now in their 50s, many have themselves had children, who have also been enrolled in the study, and grandchildren are already starting to be born. This cohort thus represents a unique opportunity to study transgenerational epigenetic phenomena, such as their studies of maternal exposures to DDT.

#### Diethylstilbestrol offspring

DES is regarded as a potential environmental endocrine disruptor by many researchers because this compound binds steroid hormone receptors and has only exogenous sources. Because exposure occurred only in relatively controlled clinic settings, cohorts of exposed individuals could in theory provide an opportunity to trace any epigenetic effects of exposure among themselves and their descendents to the restricted time periods of their pregnancies or gestation before administration to pregnant women ended in the 1970s. Exposure and comparison group data of varying quality are or could be made available from several sources, the highest quality in theory being participants in trials of DES efficacy conducted in the 1950s (Dieckmann et al. 1953; Ferguson 1953), although sample sizes were limited. Nonetheless, the numerous case–control sets, DES-exposed cohorts, and interest groups that subsequently enrolled large numbers of exposed men and women to monitor and investigate health effects of exposed individuals (Swan 2000) could prove to be valuable resources for investigation of potential epigenetic and transgenerational effects of DES among exposed individuals and their descendents. Initial proof-of-concept studies could include investigation of epigenetic marks postulated to have been disrupted by DES exposures.

#### Assisted reproductive technologies

In industrialized countries 1–4 % of neonates are now conceived by ART. Intensive surveillance of long-term health of these children has revealed somewhat increased occurrence of numerous disorders (Savage et al. 2011). However, a desirable next step in this research—determining whether ART procedures or indications for these procedures (parents’ infertility or subfertility) account for excess risk—is severely limited by paucity of data on children with a history of only one of these factors. Epigenetic mechanisms are commonly postulated for both ART and parental origins of several ART-associated conditions, including model imprinting disorders and IUGR. Therefore, new studies aiming to trace candidate epigenetic marks from relevant cells of the parent (e.g., father’s sperm) to those of affected ART-conceived children (e.g., placenta of IUGR infants or somatic cells of children with Prader-Willi syndrome) seems a logical first step to separating treatment effects from indication. More generally, studies comparing the epigenetic state of relevant cells from children conceived by ART with that of like-cell types from naturally conceived children may provide additional insights regarding conditions in early development, particularly conditions over-represented among children conceived by ART. Ongoing surveillance will doubtlessly reveal whether later onset conditions are added to this list as cohorts of children conceived by ART achieve ages at risk of diseases of adulthood. A logical extension of this surveillance would be to determine among descendents the presence of relevant phenotypes and epigenetic marks.

## Conclusions

Noteworthy experimental results, taken together, indicate that epigenetic processes could plausibly mediate effects of environmental exposures to influence disease susceptibility in mammals, since (1) specific exposures induced measurable changes in epigenetic state, (2) new epigenetic state was shown to persist after cessation of exposure, and (3) measureable phenotype was associated with exposure-related changes in epigenetic state. A fourth element, exposure-induced alteration of epigenetic marks of germ cell lineage DNA, seems necessary for transgenerational transmission of environmentally induced epigenetic changes. On this background, we interpret observational human data illustrating associations of exposures to epigenetic state and epigenetic state to disease to indicate that epigenetic mechanisms may plausibly mediate exposure effects on a wide range of human diseases.

Perhaps the greatest significance of this line of research is the potential for designing novel interventions articulated by Dolinoy and Jirtle (2008):

unlike genetic mutations, epigenetic profiles are potentially reversible. Therefore, epigenetic approaches for prevention and treatment, such as nutritional supplementation and/or pharmaceutical therapies may be developed to counteract negative epigenomic profiles.

As an example, Huang et al. (2012) have developed a therapy for reactivating the Ube3a allele that is epigenetically silenced in Angelman syndrome. However, before we begin to realize this translational potential, much research along parallel tracks needs to be conducted.

We need to better understand basic epigenetic mechanisms that operate during selected periods when the epigenome may be particularly vulnerable to environmental exposures (e.g., prenatal and early postnatal periods, childhood, puberty) as well as mechanisms that routinely maintain proper epigenetic states. We also need to understand mechanisms relevant to cumulative effects of exposure and associations with aging, since duration of exposure and aging are co-linear. A better understanding of basic mechanisms at different time points in life would guide genetic epidemiology studies in selecting environmental exposures that are more likely to affect epigenetic processes, identify the most relevant periods of exposure, identify biomarkers of exposure, and suggest biological intermediates to study.

It may be helpful to more deliberately deconstruct the steps whereby an environmental exposure may affect risk of disease via epigenetic mechanisms. For example, studies of the association of environmental exposures with selected epigenetic events as endpoints (rather than intermediates in a complex pathway) may be fruitful and will likely help us better conceive of studies to elucidate an entire pathway, from an exposure acting at least in part through epigenetic mechanisms to disease risk.

As we proceed, it will be important to consider seriously the environmental exposures to study, since we will want to select the most promising candidate exposures (those most likely to affect epigenetic processes), measuring these exposures at relevant time periods and with reasonable accuracy. Also, we cannot ignore genetic influences on epigenetic processes. We see above that studies of the epigenetic state of specific candidate genes are yielding interesting results and more such studies are warranted. Given limited knowledge of this genetic–epigenetic interface, to complement the candidate gene approach, integrative genomics approaches should be contemplated in order to combine GWAS data with epigenomic data. Clearly, statistical and bioinformatics approaches will be required to enable the efficient conduct of these analyses, especially as we expand to genome-wide scale. Similarly, research on feasible, efficient study designs, akin to current research on optimum designs for sequencing-based studies, will be needed. Finally, as with other emerging fields (e.g., the standardization of testing criteria for microsatellite instability or MSI), standardization of terminology and testing protocols will facilitate future communication of hypotheses, scientific approaches, and results.

## Abbreviations

ART: Assisted reproductive technologies
ASM: Allele-specific DNA methylation
ChIP: Chromatin immunoprecipitation
CIMP: CpG island methylator phenotype
CpG: Cytosine-phosphate-guanine dinucleotide
CRC: Colorectal cancer
DES: Diethylstilbestrol
feNO: Fractional exhaled nitric oxide
FFPE: Formalin-fixed paraffin-embedded
HDAC: Histone deacetylases
iNOS: Inducible nitric oxide synthase
IUGR: Intra-uterine growth restriction
IVF: In vitro fertilization
PBMCs: Peripheral blood mononuclear cells
PTS: Maternal smoking during pregnancy
SNP: Single nucleotide polymorphism
